# Biosensing System for Concentration Quantification of Magnetically Labeled *E. coli* in Water Samples

**DOI:** 10.3390/s18072250

**Published:** 2018-07-12

**Authors:** Anna Malec, Georgios Kokkinis, Christoph Haiden, Ioanna Giouroudi

**Affiliations:** 1 Institute of Sensor and Actuator Systems, TU Wien, Gusshausstrasse 27–29, 1040 Vienna, Austria; annmalec@gmail.com (A.M.); georgios.kokkinis@tuwien.ac.at (G.K.); christoph.haiden@tuwien.ac.at (C.H.); 2 BioSense Institute, Dr Zorana Đinđića 1, 21000 Novi Sad, Serbia

**Keywords:** magnetic labeling, magnetic microparticles, magnetophoresis, bacteria quantification, biosensing, particle tracking

## Abstract

Bacterial contamination of water sources (e.g., lakes, rivers and springs) from waterborne bacteria is a crucial water safety issue and its prevention is of the utmost significance since it threatens the health and well-being of wildlife, livestock, and human populations and can lead to serious illness and even death. Rapid and multiplexed measurement of such waterborne pathogens is vital and the challenge is to instantly detect in these liquid samples different types of pathogens with high sensitivity and specificity. In this work, we propose a biosensing system in which the bacteria are labelled with streptavidin coated magnetic markers (MPs—magnetic particles) forming compounds (MLBs—magnetically labelled bacteria). Video microscopy in combination with a particle tracking software are used for their detection and quantification. When the liquid containing the MLBs is introduced into the developed, microfluidic platform, the MLBs are accelerated towards the outlet by means of a magnetic field gradient generated by integrated microconductors, which are sequentially switched ON and OFF by a microcontroller. The velocities of the MLBs and that of reference MPs, suspended in the same liquid in a parallel reference microfluidic channel, are calculated and compared in real time by a digital camera mounted on a conventional optical microscope in combination with a particle trajectory tracking software. The MLBs will be slower than the reference MPs due to the enhanced Stokes’ drag force exerted on them, resulting from their greater volume and altered hydrodynamic shape. The results of the investigation showed that the parameters obtained from this method emerged as reliable predictors for *E. coli* concentrations.

## 1. Introduction

Microbial pathogen detection is of utmost priority for water quality control since microbial contamination threatens the health and well-being of wildlife, livestock, and human populations and can lead to serious illness and even death. Most waterborne pathogens are introduced into water supplies by human or animal urine and faeces (enteric pathogens), but they can also exist naturally in water environments as indigenous aquatic micro-organisms and the toxic compounds they produce. Traditional water monitoring techniques are typically still based on laboratory analyses of representative field-collected samples; this necessitates considerable effort and expense, and the sample may change before analysis [1]. Furthermore, currently available equipment is so large that it cannot usually be made portable. Therefore, there is an increasing demand of robust and efficient techniques of contaminants detection and of monitoring devices that save tremendous amounts of time, reagent, and sample if it is installed at contaminated sites. However, the current state of the art often fails to address same day measurements combined with multiple detection analyses and the ability to quantify the contaminants concentration in the highly diluted water samples of these water sources [2,3,4]. Moreover, major challenges that limit commercialization are instrumental complexity and large data mining capabilities.

Microfluidic systems [5,6,7,8,9,10] that use magnetic fields for performing the aforementioned tasks have provided a promising alternative that can fulfill the increasing requirements of such portable robust devices [11,12,13,14,15]. This is because magnetic fields can be well tuned and applied either externally or from a directly integrated solution in the biosensing system. In combination with these applied magnetic fields, magnetic nanoparticles are utilized. Magnetic nanoparticles have the advantage of manipulating them inside microfluidic channels by utilizing high gradient magnetic fields and their flexibility due to functionalization by means of surface modification and specific binding [16]. Specifically, these methods involve the labeling of the biological entity with magnetic particles (MPs).

In this paper, we present the proof of concept for a biosensing system able to monitor the presence of magnetically labeled *E. coli* and provide same day information on the bacterial concentration in the water sample.

## 2. Design and Methods

The system aims to detect and, most importantly, to quantify the amount of *E. coli* present within a water sample. The proposed platform is designed in such a manner so as to be applied not only for the quantification of *E. coli* (which is exclusively tested in this work) but also for any other disease-causing micro-agent, if one uses the appropriate antibodies and magnetic particles with a size relative to the analyte to be detected.

**Figure 1:**
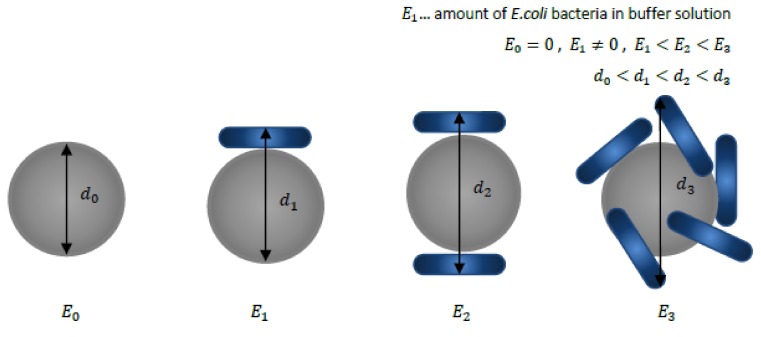
Schematic representation of the volumetric changes of a functionalized magnetic particle (MP) with respect to the number of *E. coli* bacteria attached to its surface. The amount of the attachments depends on the amount of *E. coli* bacteria present in a buffer solution. The diameter *d*_0_ corresponds to a reference diameter of a plain, not functionalized, unloaded MP without any *E. coli* bacteria attached. The variables *d*_1_, *d*_2_ and *d*_3_ indicate the sizes of the compounds formed (magnetically labelled bacteria (MLB)) when the concentration of *E. coli* bacteria within the water solution increased.

The innovative aspect of the proposed device is that it utilizes a single particle tracking system that analyzes the dynamics and volumetric changes of an MP after microorganisms are bound to its functionalized surface. The attachment of the microorganisms on the surface of the MPs is ensured by an appropriate biological binding protocol. The protocol efficiency is optimized by adjusting various parameters and conditions. Next, the measurements are conducted, provided that the labeling protocol is consistent (i.e., the amount of liquid, ratios, and handling of the sample and environmental conditions remain unchanged). Only the *E. coli* concentration is altered, which, in turn, is expected to increase/decrease the number of *E. coli* attached to the functionalized MPs (see Figure 1) and affect the particle’s dynamics.

The change in shape/volume and the resultant surface chemistry depend not only on the size/type of the attached pathogen but also on the formation of other layers/coatings arising from the procedure followed during the binding protocol. Depending on the number of bacteria attached, the behavior and the dynamics of the MLBs suspended in a liquid would change. The particle dynamics are investigated by applying an external stimulus (a magnetic field gradient) and the particle’s response is analyzed. The liquid in which the reference MPs and MLBs is suspended is static. The velocity pattern of the MLBs and the reference MPs when accelerated from the inlet to the outlet by the application of the magnetic field gradient defines the concentration of *E. coli* present in the water sample.

**Figure 2:**
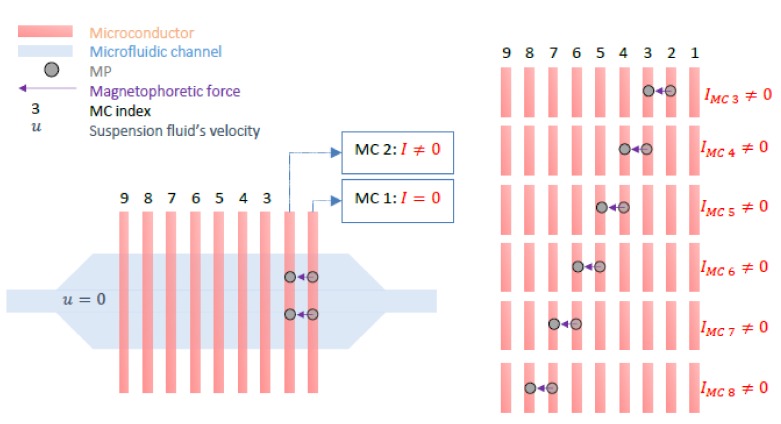
Schematic of the developed reference microfluidic channel with the integrated microconductors (MCs). A MP is manipulated from the right to the left by switching the current ON and OFF on adjacent MCs. The magnetic force is always collinear to the gradient of the magnetic field.

The bacteria quantification principle is based on the decreased MLB’s velocity due to inhibiting factors such as the Stokes’ drag force and the altered hydrodynamic shape of the initial, functionalized MP after bacteria are attached to its surface [17,18,19,20,21,22,23,24,25,26,27]. A microfluidic platform with integrated microconductors (MCs) and a polydimethylsiloxane (PDMS) channel is used to manipulate the motion of the reference MPs and that of the MLBs. The reference channel is filled with the water sample containing plain, non-functionalized reference MPs and the measurement channel is filled with the water sample containing the MLBs. Experiments were conducted provided that the suspension liquid is static (no flow conditions, *u* = 0). By controlling the direct current (DC) through the microconductors (switching it ON and OFF by means of a microcontroller), a magnetic field gradient at each conductor at a time is created and the MPs and MLBs are set in motion at the same time. The measurement begins as follows; when the MPs and MLBs reach the position where the magnetic field is stronger (i.e., it is captured on the conductor where *I* ≠ 0), the current is switched OFF [17,20,21]. This procedure is repeated for each adjacent MC and continues until the MPs and MLBs travel the entire distance from the inlet (MC 1) to the outlet (MC 9) of the chip as seen in Figure 2.

The operating principle of this microfluidic platform is described in detail in [3]. The behavior and dynamics of differently loaded MPs are observed with a video fluorescence microscope. The magnetically labeled *E. coli* bacteria are also labeled with a fluorophore for imaging purposes. The recorded video is converted to gray-scaled frames.

### 2.1. Particle Tracking Principle

**Figure 3:**
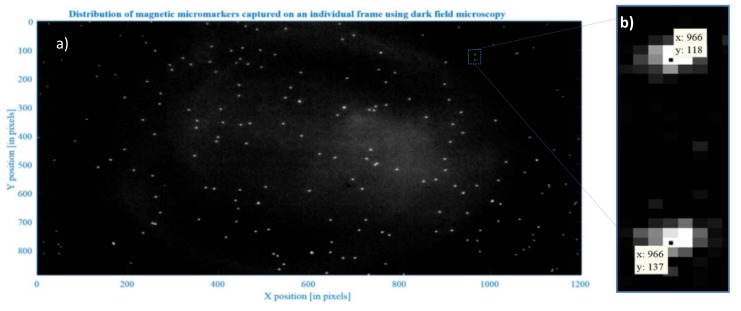
Magnetic micromarkers appear as bright spots on a dark background image. (**a**) multiple microparticles captured on a single frame *i* and (**b**) close-up for two particles with centroids assigned to each particle. For every frame, the coordinates *xi* and *yi* of the particles’ centroids are calculated.

A MATLAB script (2011b, MathWorks, Natick, MU, USA) utilizing 2D particle tracking (Crocker–Grier algorithm [28]) is used to link the exact position (centroids) of objects appearing over subsequent frames [29,30]. Based on this approach, a single frame *i* (*i* = 1, …, *N*) is processed to detect multiple bright spots (representing particles) over a dark background (here the variability in pixel intensity is used to ‘find’ a particle) (see Figure 3b). This procedure is repeated (for all the frames; *N* is the total number of all frames) giving *xi*- and *yi*-coordinates (of each individual particle’s centroid for each frame), which are then linked together to form trajectories [31].

Once particles are detected and their positions are known (for an entire sequence of video images), their locations are matched with successive and proceeding frames (i.e., it is determined which particle in a given frame most likely corresponds to the particle in the preceding frame). In order to avoid linking errors, parameters such as threshold, mask, noise length, object length, linking distance, minimum trajectory length (see Section below) must be properly adapted (i.e., when changing the magnification of an optical system or when using differently sized magnetic micromarkers these parameters must be checked again).

**Figure 4:**
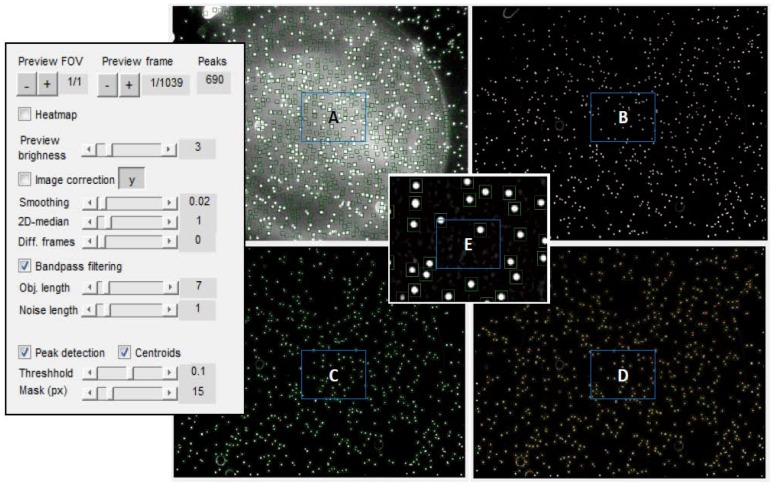
Graphical user interface (GUI) enabling preview of a selected frame for proper configuration of the detection parameters. This particular frame was taken from a video that recorded the Brownian motion of unloaded Dynabeads^TM^ M-270 Carboxylic Acid. There are 690 particles detected on this frame; (**A**) is an original video frame before noise removal by applying bandpass filtering; (**B**) is an image with increased brightness; (**C**) shows a preview with detected particles (embedded in a green squared mask); (**D**) is the preview of the image with visible particles’ centroids (multiple red points). Regular spherical shapes of particles can be seen on the zoomed picture (**E**).

**Figure 5:**
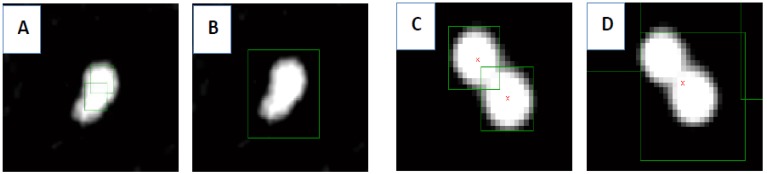
Particle detection and MP-*E. coli* complex recognition depend on proper adjustment of the detection parameters. Pictures (**A**,**B**) represent a loaded MP (MLB). Pictures (**C**,**D**) represent unloaded MPs: In (**A**), MLB is recognized as two separate objects (MP and *E. coli* are recognized separately) while, in picture (**B**), the complex is identified as a whole. In (**C**), two neighboring particles are distinguished as separate objects; on (**D**), they are marked as one. Proper adjustment of parameters must be done to obtain reliable results.

During recording of a particle’s dynamics, an important parameter is the camera frame rate. It defines a time step Δ*t* between successive frames. This parameter is given in frames per seconds (fps). Next, after selecting the desired output frames for further analysis (a combination of frames converted from multiple videos is also possible), a preview is provided (see Figure 4) to enable adjusting of parameters for a detection procedure. Proper adjustment of the parameters highlighted in Figure 4 in the graphical user interface (GUI) box on the left is crucial when recognizing an MP-*E. coli* complex as one, single complex (see Figure 5B) and not as two separate particles (see Figure 5A). At the same time, the parameters should be chosen in such a manner that two neighboring micromarkers are recognized as separate (see Figure 5C,D).

Apart from the detection parameters, other user-defined variables must be set to specify the linking procedure:

- Minimum and maximum size are introduced to specify the size range of particles whose trajectories are further analyzed (i.e., micromarker size range)
- Resolution—enables conversion of pixel units into real dimensions in micrometers; information about resolution must be provided while executing the additional scripts and calculations
- Time step Δ*t* [in seconds]—is the time between two consecutive frames and is given by the camera frame rate (for 25 fps Δ*t* = 0.04 s)
- minimum and maximum velocity—introduced to avoid the analysis of unwanted particles (e.g., foreign objects/dirt which experience different motion than the particles of interest)

**Figure 6:**
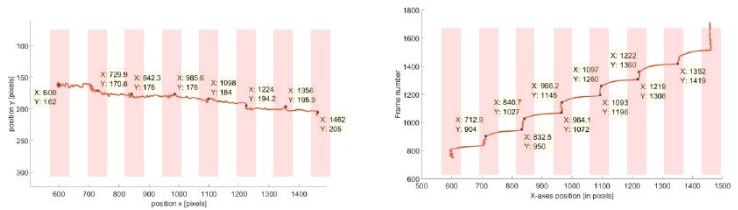
Method for representation of an MP’s motion over a sequence of frames for a single field of view (FOV). The figure on the left shows the trajectory of an individual MP with respect to *x* and *y* coordinates in (pixels). The particle is manipulated from the most left MC to the right side. Tracking starts when the MP position is at (*X*: 600, *Y*: 162) and ends on (*X*: 1462, *Y*: 205). There are visible changes in trajectory which indicate that the MP was captured at the MC (estimated *x* and *y* positions of capture are given in data boxes). Another representation of the MPs motion is shown in the figure on the right. Here, only the motion in *x*-direction (i.e., the direction of the magnetic field gradient) over the sequence of succeeding frames is plotted.

To compare the dynamics of manipulated MPs, it is necessary to further process the raw data that were obtained from the tracking software routine. Additional scripts for data representation and velocity calculations were written in MATLAB. Scripts were run for each tracking attempt and motion patterns of particles were investigated and compared. Figure 6 shows exemplary displacements of one plain MP. The direction of movement is from left to right, which can be concluded from the right-side plot. There are visible characteristic points on the trajectory that correspond to the moments when an MP was captured on the MC (i.e., time intervals when the particle is not in motion 𝑣_𝑀𝑃_ ≈ 0). Similar plots were drawn for loaded MPs.

### 2.2. Chip Design

An array of nine rectangular, parallel MCs was fabricated on a silicon wafer, below the microfluidic channels, which was wire-bonded to a printed circuit board (PCB). On top of the chip, two microfluidic channels (reference and measurement channels) made of PDMS were fabricated that were positioned perpendicularly to the MCs’ array. This configuration facilitated the flow of MPs across the MCs. The current on the MC was switched ON and OFF by means of a microcontroller. The dimensions of nine parallel rectangular MCs were selected in a way to obtain a magnetic field gradient sharp enough to move the MPs and the MLBs. The height of the MC was 1 μm (500 nm of Au and 500 nm of SiO_2_). The MCs’ dimensions were: 10 μm in width separated by 8 μm gap. The simulations as well as the explanation as to why these particular values were chosen can be found in previous works [22,32]. In short, setting an upper temperature limit at 323 K, the numerical simulation indicated a maximum current density of 9 A/m^2^. For the simulations in [22,32], a maximum current of 100 mA was applied in a sequential pattern and a time dependent solution was calculated and was reported in [22]. The MCs were fabricated on a 500 μm thick silicon wafer. The application of an insulation layer was essential since the chip’s surface was in direct contact with the biological liquid. This layer however does not inhibit the bacterial bioadhesion to the chip (silicon-based materials are very susceptible to bacterial biofouling [33]). This problem was resolved by applying a second layer (biofilm) on the chip’s surface as described below.

#### 2.2.1. Fabrication of Microconductors

The MCs were fabricated as follows: a silicon wafer served as the bottom substrate on which an image reversal photoresist was spin-coated (AZ5214, intended for lift-off-techniques). Next, Aquatar (anti-reflecting coating) was spin-coated onto the resist film. The photoresist was exposed to ultraviolet (UV) light through the 1st mask reversal-baked. This treatment caused a reaction that resulted in cross-linking the exposed areas while unexposed areas remained photoactive. The second (flood) exposure without the mask was prepared and the photoactive areas were dissolved in the developer. Afterwards, first a Ti (titanium) adhesion layer, then the Au (gold) and lastly the Cr (Chromium) films were deposited all over the surface by thermal evaporation. The unwanted parts were removed by lifting the remaining photoresist off. On the resulting microstructure, the insulating layer SiO_2_ was formed by means of plasma enhanced chemical vapor deposition (PECVD). Here, the positive photoresist AZ6624 and Aquatar were spin-coated. The structure was exposed through the designed mask. The exposed parts of the photoresist were dissolved. Afterwards, part of the passivation SiO_2_ layer was removed by oxygen etching. The remaining photoresist was stripped off. As a result, the structure consisting of the insulated MCs and the uninsulated pads was obtained.

#### 2.2.2. Microfluidic Channel

Plasma enhanced chemical vapor deposition was chosen for the microfluidic channel for its transparency, which was required for the optical microscope monitoring and software tracking. Moreover, PDMS is biocompatible and adhered to the chip’s surface without slipping and in a reversible manner. Due to its elasticity and inertness, the channel’s structure remained undamaged when applied on or removed from the chip. Therefore, this material could be used multiple times provided that any contaminants were cleaned after each operation. The dimensions of the microfluidic channel were selected in a way to be large enough to diminish the influence of unwanted factors (height = 110 µm, width in the middle where the measurement takes place = 500 µm while width of the channel from the inlet and towards the outlet = 90 µm, length = 50 mm). That is, to ignore the effects of interfacial flow turbulences due to the chemistry and the hydrophobicity of the channel’s wall. Moreover, the channel’s dimensions were adjusted to study the dynamics of multiple MPs simultaneously. The fabrication process of the microfluidic channel was as follows; on a glass wafer substrate, the negative type dry film photoresist (Ordyl SY 300 (Elga Europe, Milan, Italy)) was laminated. The mask was aligned and the light-sensitive material was exposed to the UV light. The exposed areas of the photoresist were hardened while the unexposed were dissolved in an Ordyl SY developer and removed. The obtained structure served as a mold on which liquid PDMS was slowly poured. This viscous mixture was composed of ten base units (Sylgard 184 (Sigma Aldrich, Munich, Germany)) and one unit of a curing agent and it was hardened by heating at 70 °C for 1 h on the hot plate. The obtained elastomeric PDMS channel was slowly pulled off from the mold. Lastly, the PDMS material was punched with needles in order to provide access to the channel (i.e., inlet and outlet) for the sample injection.

#### 2.2.3. Surface Modification

Surface modification was necessary to avert unwanted bacteria (or protein) interactions (e.g., adhesion) at the chip’s surface [17]. One of the strategies to avoid the adhesion of the bacteria was to apply a chemical modification on the chip with an outermost sodium alginate (SA) layer. SA exhibits a ‘brush like repulsive structure’ that keeps bacteria apart [34]. Polyethylene (POI) together with SA is layered over the insulation layer based on the layer-by-layer (LBL) electrostatic self-assembly (ESA). Treated with oxygen plasma, as described below, the SiO_2_ passivation layer becomes negatively charged, which enables attraction of positively charged polyethyleneimine (PEI). The latter one due to the cationic character attracts the anionic SA.

Specifically, the surface was functionalized by means of plasma etching which improves adhesion properties prior to coating (i.e., oxygen plasma encourages hydroxylation [35], which allows binding of the next layer via reactive −OH groups). In order to complete the surface modification steps, the chip was:

1. Rinsed with acetone, isopropanol and deionized (DI) water to remove contaminants
2. Dried for 30 min at 150 °C at a hot plate
3. Oxygen Plasma etching (here hydroxylation of the SiO_2_ passivation layer took place)
4. Dipped for 10 min in the branched, PEI 2 gL^−1^ solution that served as an adhesion promoter and rinsed with DI water
5. Dipped in sodium alginate also for 10 min 2 gL^−1^, rinsed in DI water and dried carefully with Nitrogen

This modification applies only to the surface of the chip. The PDMS channel does not have to undergo this process since: (a) MPs are not in close contact with the microfluidic channel walls and (b) the material has an intrinsic high hydrophobicity that results in inhibition of bacterial adhesion itself.

## 3. Experiments

### 3.1. Experimental Set-Up

**Figure 7:**
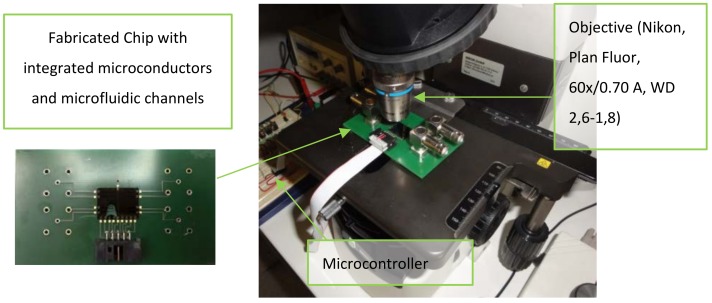
The microfluidic platform placed underneath the fluorescence microscope stage under 60× magnification, Plan Fluor objective with 2.6–1.8 mm working distance. After the measurement set with varying known *E. coli* concentrations was conducted, the new chip was sealed to the same PCB (printed circuit board) and measurements were repeated. WD: working distance.

The experimental set-up is shown in Figure 7. The microfluidic platform, placed under the optical microscope, was connected to the electronic breadboard and a DC power supply. The videos were recorded using a Nikon Camera D5100 and stored on the PC where they were later processed using the ‘particle tracking software’. In order to avoid error displacements, the camera was tightly screwed to the microscope C-mount adapter.

In these experiments, we proved the working principle, fluorescence microscopy was employed to obtain the strong image contrast between the MP-*E. coli* complex and the background and ensure the “proof of concept”, but this step is not necessary for future measurements. A high-intensity light source (high-pressure mercury vapor arc-discharge lamp) was used to evoke the sufficient photon excitation from the fluorophore (Alexa Fluor from the secondary antibody). The proper lens was selected to have high adequate magnification and a long enough working distance (here, the height of the microchannel is of relevance). A close-up of the microfluidic platform placed under the microscope can be seen in Figure 7. The same objective was used for all the measurement sets: A Plan Fluor objective with 2.6−1.8 mm working distance and 60× magnification. The field of view (FOV) was adjusted in order to eliminate the tracking errors that come from the scattering of light on the channel’s wall.

The sample is applied by pipetting a drop (2 μL) of the liquid into the channel’s inlet. Under-pressure was applied at the outlet in order to fill the channel and the excess was removed with swabs. The no-flow condition is checked under the microscope. After each measurement, the PDMS channel was rinsed with Acetone, Isopropanol and DI water and dried with Nitrogen. 

### 3.2. Magnetic Markers

For these experiments magnetic markers Dynabeads™ M-280 coated with streptavidin (ThermoFisher, Waltham, MA, USA) were used to label the biological target (wild type K-12 *E. coli* strain) by means of biotinylated polyclonal antibodies (rabbit anti-*E. coli* Abcam@ ab20640). The loading of the magnetic marker was achieved by strong noncovalent bonds between the streptavidin layer of the marker and the biotin molecules attached on the surface of the antibodies (such that the biotin-streptavidin lock-and-key coupling system can only be broken under harsh conditions: pH 4, high temperature or salt concentration).

### 3.3. E. coli Sample Preparation

K-12 wild-type *Escherichia coli* bacteria were cultured on a plastic disposable petri dish layered with a solid plain nutrient AGAR (derived from the polysaccharide agarose) with growth temperature at 37 °C and storage temperature at 3 °C. Four bacteria concentrations, (*c* = 10^3^ CFU/mL, 2 × 10^3^ CFU/mL, 3 × 10^3^ CFU/mL, 4 × 10^3^ CFU/mL), were washed three times (washing; initial suspension was centrifuged for 8 min at 4.7 × 10^3^ rpm, the supernatant was removed, the sedimented bacteria were resuspended in 1 mL 0.01M Phosphate-buffered saline (PBS)-Tween20 (0.01% *v*/*v*) and vortexed for 1 min at 10^4^ rpm). In addition, 20 mL of this *E. coli* suspension was mixed with 7 μL of the original antibody concentration and incubated for 1 h on a multiple rotator (room temperature) to yield binding. Afterwards, the sample was again washed five times and re-suspended in 100 mL of 0.01 M PBS–BSA (bovine serum albumin) (0.1% *w*/*v*). Multiple washing was conducted throughout the sample preparation so as to eliminate the risk of unspecific binding. Simultaneously, 100 mL of original concentration Dynabeads^TM^ M-280 Streptavidin (MPs) were magnetically washed and vortexed three times in 1 mL 0.01 M PBS–Tween 20 (0.01% *v*/*v*) then condensed back to 100 mL. Afterwards, the two samples were combined: 1 µL of washed MPs and 40 µL of the previously prepared complex (*E. coli*-Ab20640), the sample was left for 1 h to incubate on a multiple rotator to induce uniform biotin-streptavidin binding along the antibody-MP suspension. Next, a PBS-BSA washing buffer was added (to dilute the sample and facilitate magnetic washing) and the vial was left on a magnetic stand for approximately ≈1.5 min (during this time, MPs were attracted on the side wall towards the magnet). In the last step of the binding protocol, the supernatant was carefully discarded from the vial and the *E. coli*-loaded-MPs that were re-suspended in 100 mL PBS-BSA.

## 4. Results and Discussion

A DC power supply supplied the MCs with a constant DC voltage value of 12 V. The current in every individual MC was 53 mA. The peak force exerted on a particle is approximately 1 pN. The sample with plain, non-functionalized MPs was prepared and the MPs were additionally coupled with primary and secondary antibodies. Furthermore, the secondary antibodies were additionally labeled with a fluorophore for visualization purposes to obtain images with high contrast (bright spots over the dark background), which was essential for the tracking software.

**Figure 8:**
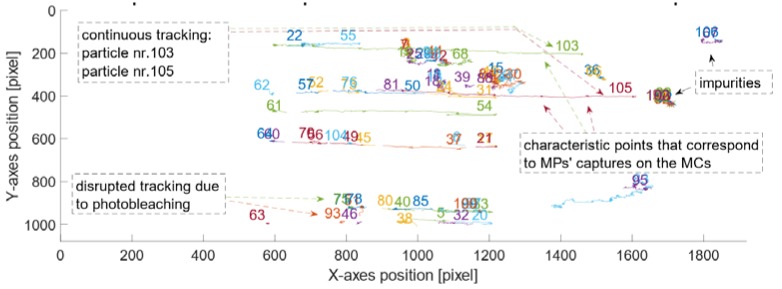
Positions for all detected and tracked particles, without *E. coli* attached to their surface, over the sequence of frames (1920 × 1080 pixels) collected at 25 fps. The particles were manipulated horizontally. There are some characteristic points visible on these trajectories. They correspond to the position of the MP as it was captured at a MC. There are over 105 trajectories detected for this sample.

Once the particles were injected in the PDMS channel and the ‘no-flow’ condition was satisfied, the manipulation started. After obtaining videos free of artifacts, the sample could be used in the software tracking procedure (see Section 2.1). Figure 8 represents all tracked particles in the reference channel, without *E. coli* attached to their surface, within 1800 frames (i.e., the centroids of bright spots that correspond to particles). Frames were collected at 25 fps (with high quality’ and ‘high sensitivity’ settings) and the frames’ dimensions were 1920 × 1080 pixels.

**Figure 9:**
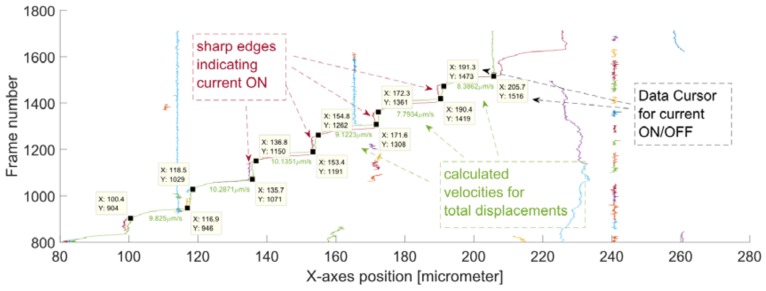
The displacement of the tracked MPs over a sequence of frames without any *E. coli* attached to their surface. There are two characteristic manipulation paths for: MP *nr*. 103 (green) and MP Nr. 105 (dark red).

The MPs were set in motion horizontally i.e., along the *x*-axes of the frame (for a width of 1920 pixels). The pixels were converted into real dimensions by applying the previously calculated resolution (*Res* = 7.2 pix/µm). The displacement of the tracked MPs (without *E. coli*) over a sequence of frames is presented in Figure 9.

From this graph, the estimated MPs’ positions, corresponding to the beginning and the end of motion, could be extracted (i.e., these are the sharp edges on the graph that indicate the current switching). From these positions (i.e., data seen in the Data Cursors in Figure 9), the mean velocities for the displacement (i.e., between two neighboring MCs) were calculated using a MATLAB script.

**Figure 10:**
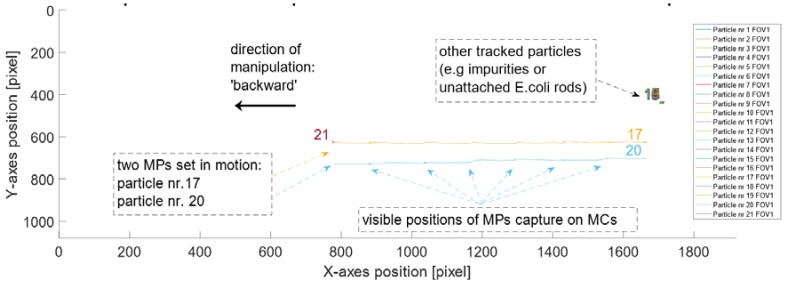
For MLBs with concentration of 2 × 10^3^ CFU/mL: Positions for all the detected and tracked MLBs over a sequence of frames (1920 × 1080 pixels) recorded at 25 fps. There are some characteristic points visible on these trajectories. They correspond to the position of the MLBs as they were captured above the MCs. There are over 21 particles (including impurities) detected for this sample. MLB Nr. 17 and MLB Nr. 20 correspond to two different MLBs that were set in motion due to the magnetic field gradient.

**Figure 11:**
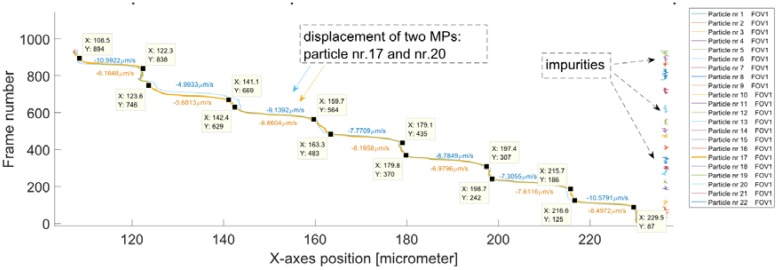
For MLBs with known concentration of 2 × 10^3^ CFU/mL: the displacement of tracked particles over the sequence of frames. There are two characteristic manipulations of MLBs: MLB Nr. 17 (yellow) and MLB Nr. 20 (blue). The rest of the tracked particles are impurities (on the right) and can be clearly differentiated from MLB.

The plots in Figure 8 and Figure 9 were drawn for all of the following samples that contained particles attached to known varying concentrations of *E. coli* (i.e., first, the plot of the positions for tracked MPs in pixels along the *x*- and *y*-axis was drawn and then the displacement of the tracked MPs over the sequence of frames). All the samples were successfully manipulated and tracked. The developed MATLAB script was used to calculate the velocities for all of the samples with varying concentrations 𝑐 = 10^3^ CFU/mL, 2 × 10^3^ CFU/mL, 3 × 10^3^ CFU/mL, 4 × 10^3^ CFU/mL. Figure 10 and Figure 11 present the plots drawn for *E. coli* concentration of 2 × 10^3^ CFU/mL.

For this sample, two MLBs were present on the recorded FOV. The outputs of the tracking routine were excellent due to the uninterrupted trajectories. There were only a few impurities detected. Such a good quality of the tracking process is a result of the high fluorescence intensity of the MLBs, sufficient sample washing and setting appropriate parameters on the software (as a result no background noise was tracked). The plots of the movement along the *x*-axis for two MLBs overlap, which is accurate because both of the MLBs are loaded with the same *E. coli* amounts.

**Figure 12:**
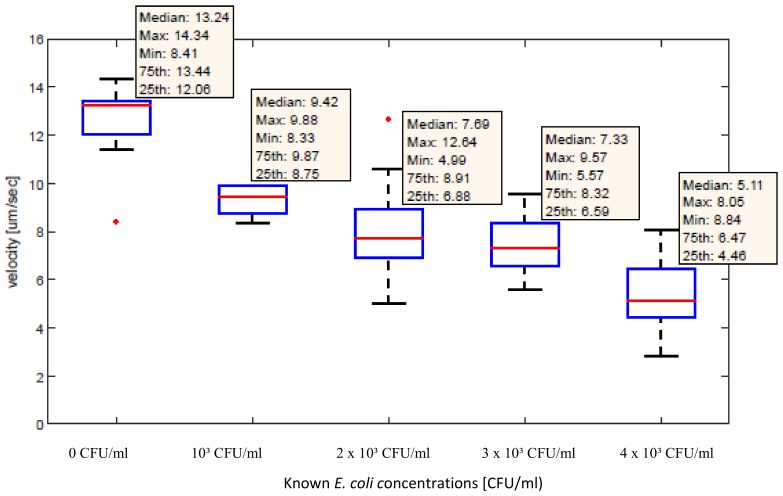
The velocities of the manipulated MPs and MLBs for different *E. coli* concentrations.

After the analysis and calculations of the velocities for all samples the data were represented in the form of a box plot (see Figure 12). All of the MPs (reference channel) and MLBs (measurement channel) were manipulated with the same current value of 53 mA. A decrease in velocity as a response to an increased *E. coli* concentration was observed.

## 5. Conclusions

The results obtained indicate that the developed biosensing system is a less complex, real-time approach for the detection of biological markers and pathogens suspended in a small volume liquid sample. In our future research, we intend to conduct experiments to determine whether the proposed technique is applicable not only for *E. coli*, but also for other bacteria as this approach has the potential to be applied not only for detection of *E. coli*, but also for a variety of microorganisms if the appropriate biological binding/labeling protocol is provided as well as for obtaining information about dynamics in life cycle of single/multiple microorganism(s) in response to condition/environmental changes. As this work was a proof of principle, a lab microscope was used to verify the feasibility of the system. Portable solutions have already been presented in [30,31] and it is the aim of future work to miniaturize the system for fluorescence single particle tracking in order for it to be transportable for in-field measurements.
